# Learning to make informed health choices: Protocol for a pilot study in schools in Barcelona

**DOI:** 10.12688/f1000research.21292.3

**Published:** 2020-06-15

**Authors:** Laura Martínez García, Pablo Alonso-Coello, Laia Asso Ministral, Clara Ballesté-Delpierre, Carlos Canelo Aybar, Carol de Britos, Ana Fernández Rodríguez, Ana Gallego Iborra, Victoria Leo Rosas, Paloma Llaquet, Ena Pery Niño de Guzmán Quispe, Giordano Pérez-Gaxiola, Carolina Requeijo, Karla Salas-Gama, Laura Samsó Jofra, Jordi Terres, Iratxe Urreta, Sarah Rosenbaum

**Affiliations:** 1 Iberoamerican Cochrane Centre (IbCC) - Sant Pau Biomedical Research Institute (IIB-Sant Pau), Barcelona, Spain; 2 CIBER of Epidemiology and Public Health (CIBERESP), Barcelona, Spain; 3 Maternal and Child Health Service, General Subdirectorate of Health Promotion, Public Health Agency of Catalonia, Barcelona, Spain; 4 ISGlobal, Hospital Clínic, University of Barcelona, Barcelona, Spain; 5 Escola Virolai, Barcelona, Spain; 6 Escola Sant Martí, Barcelona, Spain; 7 Andalusian Health Service, Malaga, Spain; 8 Paediatric Hospital of Sinaloa, Sinaloa, Mexico; 9 Epidemiology and Public Health Department, Hospital de la Santa Creu i Sant Pau, Barcelona, Spain; 10 Institut Escola Antaviana, Barcelona, Spain; 11 Clinical Epidemiology and Research Unit, University Hospital of Donostia, Donostia, Spain; 12 Centre for Informed Health Choices, Norwegian Institute of Public Health, Oslo, Norway

**Keywords:** Children’s health, critical thinking, evidence-based medicine, health education, health promotion, public health.

## Abstract

**Introduction:** The Informed Health Choices (IHC) project has developed learning resources to teach primary school children (10 to 12-year-olds) to assess treatment claims and make informed health choices. The aim of our study is to explore both the students’ and teachers’ experience when using these resources in the context of Barcelona (Spain).

**Methods:** During the 2019-2020 school year, we will conduct a pilot study with 4 ^th^ and 5 ^th^-year primary school students (9 to 11-year-olds) from three schools in Barcelona. The intervention in the schools will include: 1) a workshop with the teachers, and 2) lessons to the students. The data collection will include: 1) assessment of the IHC resources by the teachers before the lessons, 2) non-participatory observations during the lessons, 3) semi-structured interviews with the students after a lesson, 4) assessment of the lessons by the teachers after a lesson, 5) treatment claim assessment by the students at the end of the lessons, and 6) assessment of the IHC resources by the teachers at the end of the lessons. We will use *ad hoc* questionnaires and guides to register the data. We will perform a quantitative and qualitative analysis of the data to explore understandability, desirability, suitability, usefulness, facilitators and barriers of the resources. The most relevant results will be discussed and some recommendations on how to use, how to adapt (if needed), and how to implement the IHC resources to this context will be agreed. The findings of the contextualization activities could inform the design of a cluster-randomised trial, to determine the effectiveness of the IHC resources in this context prior to scaling-up its use.

**Ethical considerations:** The study protocol has obtained an approval exemption from the Ethics Committee of the Hospital de la Santa Creu i Sant Pau (Barcelona, Spain).

## Introduction

In our day-to-day, we hear and make claims about treatments that can improve or worsen our health (“treatment” can be defined broadly as any action to improve or maintain the health of individuals). Claims we make, or are exposed to, may be about therapeutic interventions (take drugs, undergo surgery or use medical devices), changes in lifestyle (follow dietary guidelines, do exercise), interventions involving alternative medicine (use medicinal herbs), public health or environmental interventions, or changes in how health care is provided, funded or managed ^1, 2^.

Many of these claims, regardless of whether they are well-intentioned or driven by various interests, can be wrong, inadequate or untrustworthy ^3^. When people make decisions based on untrustworthy treatment claims, or when they ignore trustworthy claims, they may harm their health and use resources inadequately ^3^.

In order for people to make informed health choices, they need to be able to obtain, process and understand the relevant health information (health literacy) and use that information from a critical perspective (critical thinking) ^4– 6^. Unfortunately, many people lack that ability. A European survey showed that 58.3% of the Spanish population has a limited level of health literacy ^7^.

### Informed Health Choices project

The main objective of the Informed Health Choices (IHC) project is to teach people to assess treatment claims and make informed health choices.

The IHC project has a focus on enabling people to learn these skills at a young age and began their first work in developing learning resources for primary school children (10 to 12-year-olds) from low-income countries (Uganda) ^8^. There are several reasons the IHC project started with primary school children: 1) children can learn about fair comparisons (controlled research) and critical appraisal (in some countries, teaching these basic capabilities is already part of the curriculum) ^9, 10^; 2) primary school interventions can reach a large population group, before many of them leave school ^11^; 3) compared to adults, children have more time to learn and show less resistance to change with regard to their beliefs, attitudes or behaviours ^12^; 4) teaching children to think critically improves their academic performance ^13^; and 5) learning how to think critically about claims about treatment effects can help them, once they become adults, to make decisions about their health and to contribute, as citizens or as health decision-makers, to develop and implement health policies ^14^. In addition, the IHC project focused on the child population of low-income countries because making informed health choices can contribute to a more efficient use of resources in contexts with higher social and economic inequality ^8^.

The IHC Working Group has developed several resources to help people understand the differences between trustworthy and untrustworthy health claims, and how to use reliable information to make informed health choices ^8^. The main resources are: 1) key concepts, 2) learning resources, and 3) a tool to evaluate the ability to assess treatment claims.

***IHC key concepts.*** Using the principles of a spiral curriculum, the IHC Working Group has compiled a list of concepts that individuals need to understand and apply when assessing claims about treatment effects and making health choices ^3, 15^.

The list of concepts is reviewed and updated periodically. The list currently includes 44 concepts divided into three capability groups: 1) identify when the treatment claim has an untrustworthy basis, 2) recognise when evidence from comparisons of treatments is trustworthy and when it is not, and 3) make well-informed choices about treatments. Table 1 shows the list of key concepts ^13^.

***IHC learning resources.*** Using a human-centred design approach ^16– 18^, the IHC Working Group has produced various learning resources (IHC resources) to teach children and their families to understand and apply some of the key concepts ^8^.

**Table 1.** List of key concepts from the Informed Health Choices project ^13^.

**1. Beware of treatment claims like these:** We hear claims about the effects of treatments all the time. Many of these are not trustworthy. When you hear someone use one of these reasons to support a claim about the effects of a treatment, you should beware and ask where the evidence is.		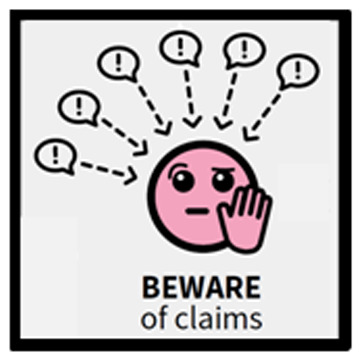
**1.1. Beware of claims that are too good to be true**	1. “100% safe!” *	
	2. “100% effective!”	
	3. “100% certain!”	
**1.2. Beware of claims based on faulty logic**	4. “Treatment needed!”	
	5. “It works like this!”	
	6. “Associated with!”	
	7. “Real world data!”	
	8. “No comparison needed!” *	
	9. “A study shows!” *	
	10. “Old is better!” *	
	11. “New is better!” *	
	12. “More is better!”	
	13. “Early is better!”	
	14. “Personalised medicine!”	
**1.3. Beware of claims based on trust alone**	15. “As advertised!” *	
	16. “It worked for me!” *	
	17. “Recommended by experts!” *	
	18. “Peer reviewed!”	
**2. Check the evidence from treatment comparisons** A treatment has to be compared to something else to know what would happen without the treatment. For treatment comparisons to be FAIR, the only important difference between comparison groups should be the treatments they receive. Unfair treatment comparisons and unsystematic summaries of treatment comparisons can be misleading. The way that treatment effects are described can also be misleading.		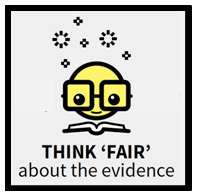
**2.1. Don’t be misled by unfair comparisons!**	19. Dissimilar comparison groups *	
	20. Indirect comparisons	
	21. Dissimilar attention and care	
	22. Dissimilar expectations or behaviours *	
	23. Dissimilar assessment of outcomes	
	24. Unreliable assessment of outcomes	
	25. Lots of people not followed-up	
	26. Outcomes counted in the wrong group	
**2.2. Don’t be misled by unreliable summaries of** **treatment comparisons!**	27. Unsystematic summaries	
	28. Selective reporting	
	29. Unfounded assumptions	
**2.3. Don’t be misled by how treatment effects are** **described!**	30. Just words	
	31. Relative effects	
	32. Average effects	
	33. Few people or events *	
	34. Subgroup analyses	
	35. Statistically significant	
	36. No confidence interval	
	37. No evidence	
**3. Make well-informed treatment choices** Deciding what to do requires judgements about the relevance of the evidence, how important the good and bad outcomes are to you, and how sure you can be about the treatment effects.		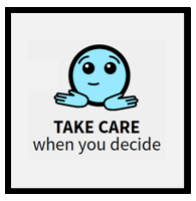
**3.1. What is the problem and what are the options?**	38. What is your health problem and what are your options?	
**3.2. Is the evidence relevant?**	39. What outcomes matter to you?	
	40. Are the people (or animals) very different from you?	
	41. Are the treatments different from those available to you?	
	42. Are the circumstances different from yours?	
**3.3. Do the advantages outweigh the** **disadvantages?**	43. Do the advantages outweigh the disadvantages for you? *	
	44. How sure are you about the treatment effects?	

* The 12 concepts included in the learning resources of the IHC project for primary school children. The IHC Key Concepts' explanations are available from the “That’s a Claim” website. This table has been reproduced with permission from Oxman *et al.* (Box 3) ^13^.

**Figure 1.**
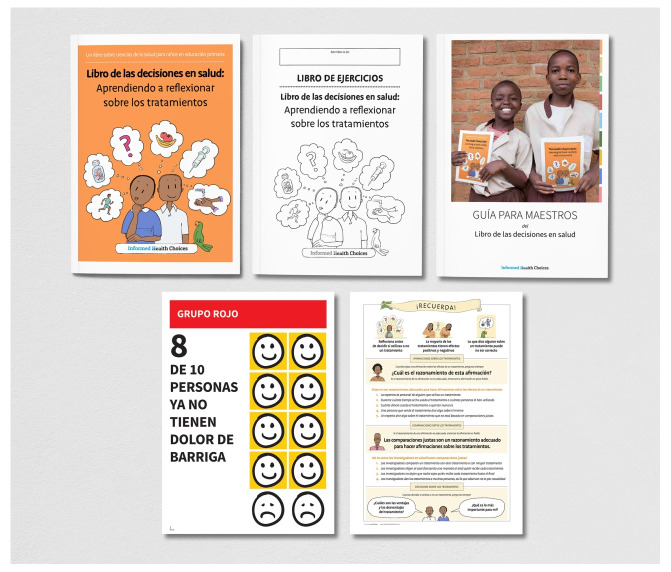
Learning resources from the Informed Health Choices project for primary school children translated into Spanish.

The following resources were produced for primary school children (10 to 12-year-olds): a book (that includes and explains 12 key concepts), an exercise book, a teachers’ guide, some activity cards, a poster and a song ( Figure 1) ^8, 19^. The book tells a story, narrated as a comic, about a brother and a sister, John and Julie, who know two teachers and health researchers, professor Compare and professor Fair. The professors teach John and Julie: 1) what questions they should ask when someone says something about a treatment; 2) what questions health researchers ask to find out more about treatment effects; and 3) what questions they should ask when deciding to use a treatment or not ^19^.

The effect of the resources was assessed in a cluster randomised trial conducted in Uganda ^14^. In the trial, 120 schools were assigned randomly to receive the intervention with the resources (60 schools, 76 teachers and 6,383 children) or not receive it (60 schools, 67 teachers and 4,430 children) ^14^. The study showed that the children who used the resources improved their ability to assess treatment claims in comparison with the group without resources (69% of the children who use the learning resources got a passing score *vs.* 27% of children in the control group) ^14^. A follow-up study one year later showed that children retained this knowledge and, in fact, the proportion of children with a passing score increased from 69% to 80% ^20^.

Additionally, the IHC project team developed and evaluated a podcast with several episodes for parents (that introduce and explain nine key concepts) ^8, 18, 21, 22^.

***Tool to evaluate the ability to assess treatment claims.*** The IHC Working Group has created a database with questions to assess people’s understanding and ability to apply the key concepts; the CLAIM Evaluation Tools ^23^. Each question is based on a scenario that involves a claim about a treatment. There are two types of questions: 1) individual multiple-choice questions and 2) several true or false statements ^23^.

This tool is a flexible resource, since people may design a questionnaire according to the key concepts that they wish to evaluate, selecting the questions that are most relevant for their objectives ^19^. For example, teachers can design questionnaires to assess children, and researchers can design questionnaires to assess interventions or to describe a population’s ability to make informed health choices ^23^. All the questions have been designed to be answered by children over 10 years of age as well as by adults ^23^. The CLAIM Evaluation Tools can be found on the Testing Treatments international website.

In the previously cited trials that assessed the effect of the IHC resources, the researchers used questions from this database ^14, 22^.

### Contextualization of the Informed Health Choices project

The IHC resources have proven to be effective in the Ugandan trial, but it is still unknown whether they may be useful in other contexts ^24^. Different working groups from more than 20 countries are adapting, or planning adaptation, of the IHC resources to their context ^25, 26^.

The IHC Working Group has proposed the following contextualization activities to explore how these resources can be used in a context different from the one that they were originally designed for: 1) context analysis, 2) translation of the resources, 3) pilot study, 3) content adaptation, 5) resource production, and 6) validation of the tool to assess treatment claims ^24^.

Currently, we do not have any specific learning resources to teach primary school children to think critically about their health in the context of Barcelona (Spain). The working group from the Iberoamerican Cochrane Centre (CCIb) - Sant Pau Biomedical Research Institute (IIB Sant Pau) has translated the IHC resources into Spanish Spanish based on methods proposed by the IHC Working Group ^27^. The Spanish IHC resources were included on the IHC website. The next step is to explore how to use and, if needed, how to adapt the IHC resources in this context.

## Objectives

### Primary objective

•    Explore the students’ and teachers’ experience when using the learning resources of the IHC project in the context of Barcelona (Spain).

### Secondary objectives

•    Explore potential changes to the IHC resources to adapt them to this context.

•    Explore the feasibility of implementing the IHC resources in this context.

•    Evaluate the ability of the students to assess treatment claims and make informed health choices after using the IHC resources in this context.

## Methods

During the 2019–2020 school year, we will conduct a pilot study with 4th and 5th-year primary school students (9 to 11-year-olds) from three schools in Barcelona, based on methods proposed by the IHC Working Group ^28^. Table 2 shows and describes the different steps of the pilot study.

**Table 2.** Pilot study tasks.

Tasks	Participants	Activities
1. Establishment of the IHC-Barcelona Working Group		
**1.1. Establishment of the coordination** **group**	- Researchers	Group responsible for planning, coordinating and monitoring the different steps of the pilot study.
**1.2. Establishment of the advisory group**	- Researchers - Teachers - Paediatricians - Student representatives - Family representatives - Education and health stakeholders - Translators	Group responsible for reviewing and advising during the development of the different steps of the pilot study.
2. Protocol development		
**2.1. Protocol development**	- IHC-Barcelona Working Group	Develop and publish the pilot study protocol. Request the approval of Ethics Committee of the Hospital de la Santa Creu i Sant Pau (Barcelona, Spain).
3. Preparation of the activities in the schools		
**3.1. Selection of the schools**	- Coordination group	Select three schools in Barcelona (convenience sample).
**3.2. Selection of the students and the** **teachers**	- Coordination group	Select 4 ^th^ and 5 ^th^-year primary school students (10 to 11-year-olds) and teachers.
**3.3. Introduction of the pilot study to** **the families**	- Researchers - Teachers	Introduce the IHC project and the pilot study in a meeting with the families (first meeting of the school year and/or specific meeting about the project).
**3.4. Compilation of the informed** **consent forms**	- Teachers	Request the families and the teachers to give their informed consent (Extended data 1, 2 and 3) ^31^.
**3.5. Delivery IHC resources to the** **schools**	- Coordination group	Send a book for each student. Send a book, a teachers’ guide, activity cards, and a poster for each teacher.
4. Intervention in the schools		
**4.1. Workshop with the teachers**	- Researchers - Teachers	Introduce and review the IHC project, the pilot study, and the IHC resources with the teachers (Extended data 5) ^31^.
**4.2. Lessons to the students**	- Students - Teachers	Teach students to assess treatment claims and make informed health choices using the IHC resources.
5. Data collection		
**5.1. Assessment of the IHC resources** **by the teachers before the lessons**	- Teachers	Explore the teachers’ initial perception of the IHC resources (Extended data 6) ^31^.
**5.2. Non-participatory observations** **during the lessons**	- Researchers	Assess (objectively) the degree of implementation of the IHC resources and explore the students’ experience when using the IHC resources (Extended data 7) ^31^.
**5.3. Semi-structured interviews with** **the students after a lesson**	- Students - Researchers - Teachers	Explore the students’ experience when using the IHC resources (Extended data 8) ^31^.
**5.4. Assessment of the lessons by the** **teachers after a lesson**	- Teachers	Assess (self-reportedly) the degree of implementation of the IHC resources and explore the teachers’ experience when using the resources (Extended data 9) ^31^.
**5.5. Treatment claim assessment by the** **students at the end of the lessons**	- Students	Evaluate the ability of the students to assess treatment claims and make informed health choices after using the IHC resources in this context (the questionnaire is accessible upon request from the Testing Treatments website to preserve the validity of the questions).
**5.6. Assessment of the IHC resources** **by the teachers at the end of the** **lessons**	- Teachers	Explore the teachers’ final experience when using the IHC resources and compare their initial perception with the final experience (Extended data 10) ^31^.
6. Data analysis		
**6.1. Data analysis**	- Researchers	Quantitative and qualitative analysis of the data.
7. Formulation of the recommendations		
**7.1. Formulation of the recommendations**	- IHC-Barcelona Working Group	Suggest and agree some recommendations on how to implement the IHC resources in this context.
8. Dissemination of the results		
**8.1. Dissemination of the results**	- IHC-Barcelona Working Group	Publish in a peer-reviewed journal, publish in several internet resources and introduce to the different users of interest.

### Participants

***Establishment of the IHC-Barcelona Working Group.*** We will establish a “coordination group” to lead and coordinate the pilot study and to ensure it is completed according to the established work plan. We will establish a multidisciplinary “advisory group” (researchers, teachers, paediatricians, student representatives, family representatives, education and health stakeholders, and translators) to review and advise on the development of the different steps of the pilot study.

We will aim for profile representativeness of the IHC-Barcelona Working Group members. We will identify researchers from CIBER of Epidemiology and Public Health (CIBERESP) and expert colleagues; teachers, student representatives, and family representatives from selected schools; paediatricians from Asociación Española de Pediatría de Atención Primaria (AEPap); education and health stakeholders from Catalan Education and Health Departments; and translators who participated in the IHC resources translation into Spanish. Potential members will be contact and invite to participate by email. We will request and register the conflicts of interest of all the members of the IHC-Barcelona Working Group.

***Selection of the schools.*** To achieve the objective, we will select a convenience sample of three schools in Barcelona ^29^. The IHC-Barcelona Working Group reach a consensus on eligibility criteria of the schools: 1) schools included in the school directory from the Department of Education from the Government of Catalonia (2018–2019); 2) schools that have participated in a health promotion programme (2016–2017) ^30^; and 3) schools that take part in the initiative *Escola Nova 21* (alliance of schools and civil society institutions for an advanced education system, carried out between 2016 – 2019, and responding to United Nations and UNESCO’s call for the participation of all sectors in an inclusive process to make possible the education paradigm shift). We will also take into consideration whether the schools include students that are representative of the neighbourhood, if they are in different neighbourhoods of the city, and their type of funding (two public schools and one private or charter school).

***Selection of the students and teachers.*** We will select 4 ^th^ and 5 ^th^-year primary school students (9 to 11-year-olds) in all the lines from the selected schools (in this context, the number of lines means the number of student groups per academic level). We expect to include a convenience sample of approximately 150 students (25 students per class * two lines per school * three schools). We will request written informed consent from the families (Extended data 1 and 2) ^31^.

We will select one teacher from every 4 ^th^ or 5 ^th^ year class in the selected schools. We expect to include six teachers (one teacher per class * two lines per school * three schools). The profile of the participatory teachers, as well as the subject where the lessons will be included (for example, in Science, Ethics or even Spanish) will depend on the education plan and the availability of the resources in each school. We will request informed consent from the teachers (Extended data 1 and 3) ^31^.

### Intervention in the schools

The intervention in the schools will include: 1) a workshop with the teachers, and 2) lessons to the students (Extended data 4 provides a description of the intervention using the TIDieR checklist) ^31, 32^. Each of the activities is summarised below:

1. Workshop with the teachers

The objective is to introduce and review the IHC project, the pilot study, and the IHC resources with the teachers.
Before the workshop, a paper copy of the IHC resources translated into Spanish will be sent to the teachers for their review. During the workshop, a researcher from the IHC-Barcelona Working Group will introduce the IHC project and the pilot study. In addition, a mock lesson will be taught as an example (previously selected by the teachers). Finally, a teacher from each school will explain the plan to teach the lessons to the student body. The workshop will last approximately five and a half hours ( Table 3; Extended data 5) ^31^.

**Table 3.** Pilot study variables.

Variables	Assessment of the IHC resources by the teachers before the lessons	Non- participatory observations during the lessons	Semi- structured interviews with the students after a lesson	Assessment of the lessons by the teachers after a lesson	Treatment claim assessment by the students at the end of the lessons	Assessment of the IHC resources by the teachers at the end of the lessons
1. Questionnaire identification	X	X	X	X	X	X
2. Students’ experience with the IHC resources (understandability, desirability, suitability, and usefulness)	X	X	X	X		X
3. Teachers’ experience with the IHC resources (understandability, desirability, suitability, and usefulness)	X	X		X		X
4. Technique used to teach the lesson		X		X		
5. Facilitators and barriers to teach the lesson		X		X		
6. Examples of treatment claims	X	X	X			
7. Suggestions to improve the lesson			X	X		
8. Questions		X	X	X		
9. Comments	X	X	X	X		X
10. Treatment claim assessment					X	

2. Lessons to the students

The objective is to teach students to assess treatment claims and make informed health choices using the IHC resources.
The IHC resources were designed to be used over nine weeks, with one double period (80 min) per week, during a single term, and one hour to complete the test at the end of the term ^14^. In the pilot study, we will require to read and discus the story during each lesson. Although the teacher will be able to adapt the lessons to their students depending on the education plan of each school. The criteria that the teachers must take into consideration are:
Continuity of lessons (number of lesson/week and number of weeks)
Duration of lessons (number of minutes/lesson)
Completion of some or all activities and/or exercises proposed in the lessons
Resource format (Spanish and/or English, printed and/or digital)
Completion of extra activities
The teachers will reach an agreement with the IHC-Barcelona Working Group regarding their proposal for adaptation.

### Data collection

The data collection will include: 1) assessment of the IHC resources by the teachers before the lessons, 2) non-participatory observations during the lessons, 3) semi-structured interviews with the students after a lesson, 4) assessment of the lessons by the teachers after a lesson, 5) treatment claim assessment by the students at the end of the lessons, and 6) assessment of the IHC resources by the teachers at the end of the lessons. Each of the activities is summarised below:

1. Assessment of the IHC resources by the teachers before the lessons

The objective is to explore the teachers’ initial perception of the IHC resources.
We will explore the teachers’ initial perception of the IHC resources using an *ad hoc* self-administered questionnaire after the workshop. The questionnaire will include: teacher’s impression of the students’ expected experience with the IHC resources (understandability, desirability, suitability, and usefulness), the teachers’ experience with the IHC resources (understandability, desirability, suitability, and usefulness), examples of treatment claims, and comments ( Table 3; Extended data 6) ^31^..

2. Non-participatory observations during the lessons

The objectives are to assess (objectively) the degree of implementation of the IHC resources and explore the students’ experience when using the IHC resources.
A researcher from the IHC-Barcelona Working Group will make the non-participatory observations during the lessons. For convenience, each lesson will be observed in two classes (18 observations). Which lesson is going to be observed in each class will be assigned randomly. Each non-participatory observation will be audio-recorded and transcribed. The researcher will register his or her observations in an *ad hoc* guide that will include: researcher’s impression of the students’ and teachers’ experience with the IHC resources (understandability, desirability, suitability, and usefulness), technique used to teach the lesson, the facilitators and barriers to teach the lesson, examples of treatment claims, questions, and comments ( Table 3; Extended data 7) ^31^. Another researcher will check the notes with the recorded audios. The two researchers will resolve potential disagreements by discussion, and if necessary, by consulting a third researcher.

3. Semi-structured interviews with the students after a lesson

The objective is to explore the students’ experience when using the IHC resources.
A researcher from the IHC-Barcelona Working Group will hold, with the support of a teacher, semi-structured individual interviews with a selection of students after a lesson. For convenience, two interviews will be held per lesson (18 interviews). Which student is going to be interviewed in each class will be assigned randomly (using the alphabetical attendance sheet). In the event that any of the selected students does not wish to participate, the next student will be selected from the list. Each interview will last approximately 30 minutes, and its audio will be recorded and transcribed. The researcher will hold the semi-structured interview using an *ad hoc* guide that will include: the students’ experience with the IHC resources (understandability, desirability, suitability, and usefulness), examples of treatment claims, suggestions to improve the lesson, questions, and comments ( Table 3, Extended data 8) ^31^. Another researcher will check the notes with the recorded audios. The two researchers will resolve potential disagreements by discussion, and if necessary, by consulting a third researcher.

4. Assessment of the lessons by the teachers after a lesson

The objectives are to assess (self-reportedly) the degree of implementation of the IHC resources and explore the teachers’ experience when using the resources.
After teaching each lesson, the teachers will assess it in an *ad hoc* self-administered questionnaire. The questionnaire will include: teacher’s impression of the students’ experience with the lesson (understandability, desirability, suitability, and usefulness), the teachers’ experience with the lesson (understandability, desirability, suitability, and usefulness), the technique used to teach the lesson, the facilitators and barriers to teach the lesson, suggestions to improve the lesson, questions, and comments ( Table 3, Extended data 9) ^31^.

5. Treatment claim assessment by the students at the end of the lessons

The objective is to evaluate the ability of the students to assess treatment claims and make informed health choices after using the IHC resources in this context.
After completing all the lessons, the students will take a self-administered test (CLAIM questionnaire test) to evaluate their ability to apply the concepts discussed during the lessons. The test will include 24 questions (15 multiple-choice questions and nine true or false statements) from the CLAIM Evaluation Tools ( Table 3; the questionnaire is accessible upon request from the Testing Treatments website to preserve the validity of the questions). The evaluation will be in Spanish (even if the resources were used in English), on a paper copy, and with a duration of approximately 60 minutes.

6. Assessment of the IHC resources by the teachers at the end of the lessons

The objectives are to explore the teachers’ final experience when using the IHC resources and compare their initial perception with the final experience.
After completing all the lessons, we will explore the teachers’ final experience with the IHC resources using an *ad hoc* self-administered questionnaire. The questionnaire will include: teacher’s impression of the students’ experience with the IHC resources (understandability, desirability, suitability, and usefulness), the teachers’ experience with the IHC resources (understandability, desirability, suitability, and usefulness), and comments ( Table 3; Extended data 10) ^31^.

### Data analysis

***Quantitative analysis.*** We will perform a descriptive analysis of the categorical variables (absolute and relative frequencies), and the continuous variables (mean and standard deviation or median and range).

With regard to the treatment claim assessment by the students, we will show the mean score and the standard deviation, the proportion of the students with a passing score (basic knowledge of the concepts and how to apply them, 13 points or more over 24), and the proportion of the students with a high score (clear knowledge of the concepts and how to apply them, 20 points or more over 24) ^33^.

***Qualitative analysis.*** We will register in an excel sheet the feedback from: 1) the initial assessments by the teachers, 2) the non-participatory observations, 3) the semi-structured interviews with the students, 4) the assessment of the lessons by the teachers, and 5) the final assessments by the teachers.

We will perform a thematic analysis based on the categories previously used in the IHC project (seriousness, user experience, facilitators and barriers, and potential changes) ( Table 4) ^28, 34^. One researcher will identify, codify, and summarise the feedback using these categories and search for emerging categories; another researcher will check the codification. They will discuss and review the definitions and limits of each category. Finally, using the summarised data, they will explore the nature of the phenomena (understandability, desirability, suitability, usefulness, facilitators and barriers) and the possible explanations of the results.

**Table 4.** Categories of the qualitative analysis for the pilot study ^28, 34^.

Categories		Description
Seriousness for the user		
Severe problem	**XXX**	Issues associated with incorrect (or a lack of) understanding, critical errors, severe lack of interest, or any issue that may result in abandoning the whole exercise, task or lesson
Serious problem	**XX**	Issues associated with frustration, unnecessarily slow use, or deviation from the lesson guide/plan but that are either resolved or do not interfere with the learning/teaching/use in a critical way
Minor problem	**X**	Minor or cosmetic issues that probably don’t have consequences for use, such not liking some detail in the drawing
Positive feedback with changes	**00**	Praise where we should consider changes in the resources
Positive feedback without changes	**0**	Praise that do not involve a change in the resources
Suggestions	**i**	A suggestion made by the participant
User experience		
Understandability	Easy for participant to comprehend (content) and recognize (type of product)	
Desirability	Something the participant wants, likes, or has a positive emotional response	
Suitability	Something the participant feels is for “someone like me”, is suitable for use in her context	
Usefulness	Helpful to participant in achieving her goals/tasks/needs	
Facilitators and barriers		
**Teachers**	Profiles and competences	Teacher’s education and experience in relation to the lessons being taught
	Understanding of the content being taught	Teachers’ understanding of the context
	Sufficient training	The extent to which the teachers received sufficient training in teaching the lessons
	Self-efficacy	Teacher’s confidence in teaching the lessons
	Fit to the teacher’s teaching style and context (e.g., class size)	Teachers’ comfort or ability to adapt the instructions to their style and context
	Attitudes	Teachers’ attitude towards new resources (change), science, critical thinking and independent thinking by the student body (or their role as authorities in the classroom)
	Beliefs	Teachers’ beliefs about the methods or content (e.g., what treatments work or the concepts)
	Emotions	Teachers’ emotions, such as stress or anxiety
	Motivation	Teachers’ motivation to teach the material
	Positive learning environment	Teachers’ ability to create a positive learning environment; for example, encourage discussion, respond positively to questions, engage students
**Students**	Literacy	Students’ ability to read and understand the material
	Attendance	Students’ attendance or reasons for poor attendance (e.g., long distance to school or inability to pay school fees)
	Motivation to learn	Students’ motivation to learn the new material
	Attitudes	Students’ attitudes towards learning, towards authorities, towards science, towards critical thinking
	Beliefs	Students’ beliefs about the content (e.g., what treatments work or the concepts)
	Home environment	The extent to which the student’s home environment encourages or discourages learning from the lessons
	Differentiated instruction	The extent to which students different learning needs are met
	Peer influence	Positive or negative attitudes of other students towards the material
**Learning resources**	Value of the material	The extent to which the materials are valued by the teachers and students
	Compatibility with the curriculum	The extent to which the resources fits with the rest of the curriculum and how it is taught
	Appropriateness of the material	The extent to which the resources are relevant, challenging and engaging
	Credibility of the material	The extent to which the teachers and students perceive the resources as credible
**School system and** **environment**	Time constraints	The extent to which there is sufficient time to accommodate introducing the new material
	Competing priorities	The extent to which other priorities for the school, teachers or students limit introducing the resources (e.g., preparing for exams)
	School organisation and management	The extent to which the school provides an environment that supports adoption of new subjects, resources and teaching methods
	School resources, particularly human resources	The extent to which the school has adequate resources to introduce the new resources (e.g., human resources, student/teacher ratio, teacher workload, classroom space and classroom resources, such as blackboards and acoustics)
	Attitudes and beliefs of head teacher and other teachers	Attitudes or beliefs of colleagues that influence the teacher’s interest in and ability to teach the material
	Parent and community involvement	Parents’ attitudes towards the new resources or how things are done at the school
	Regulations	Regulations (e.g., Ministry of Education policies and regulations) that affect introducing the new material
	Political environment	Elements of the political environment that affect introducing the new material; for example, authoritarianism or teacher strikes
	Bureaucracy	Bureaucratic arrangements that delay or limit introduction of the new materials, or facilitate introducing them
	Incentives and disincentives	Incentives or disincentives to introduce the new resources for teachers or head teachers
Potential changes		
Dramatic changes	Involve creating new IHC resources	
Major changes	Involve changing the IHC resources drawings	
Minor changes	Involve changing the IHC resources text	

The ‘Facilitators and barriers’ section of this table has been reproduced with permission from Nsangi *et al.* ( Table 1) ^34^.

### Formulation of the recommendations

The IHC-Barcelona Working Group will discuss the most relevant results from the qualitative analysis. They will reach a consensus on the potential changes of the IHC resources (dramatic changes, major changes, or minor changes) ( Table 4). Finally, they will suggest and agree on recommendations - both for practice and research purposes - on how to use, how to adapt (if needed), and how to implement the IHC resources to this context.

### Dissemination of the results

The dissemination activities of the pilot study results will include: 1) publication in a peer-reviewed journal, 2) publication in several Internet resources (for example, related web pages, electronic bulletins and social media), and 3) introduction to the different users of interest (researchers, teachers, paediatricians, student representatives, family representatives, education and health stakeholders, and translators) in conferences, workshops and meetings. The implementation activities will include: 1) offering support to the schools that have participated in the pilot study and that are interested in including the IHC resources in the following school years, 2) giving support to other schools that are interested in including the IHC resources in their education plan.

### User participation

Representatives from all the different areas of interest (researchers, teachers, paediatricians, student representatives, family representatives, education and health stakeholders, and translators) will be invited to be members of the IHC-Barcelona Working Group.

### Ethical considerations

The study protocol has obtained an approval exemption (does not include patients, biological specimens or clinical data) from the Ethics Committee of the Hospital de la Santa Creu i Sant Pau (Barcelona, Spain) ^35^. We will inform participants about the pilot study and will request their written informed consent (Extended data 1–3) ^31^. If a family does not want to participate, the student will participate in the lessons as a curricular activity but will not participate in any of the study data collection activities. We will anonymise the data removing participant and school name.

### Study status

**Figure 2.**
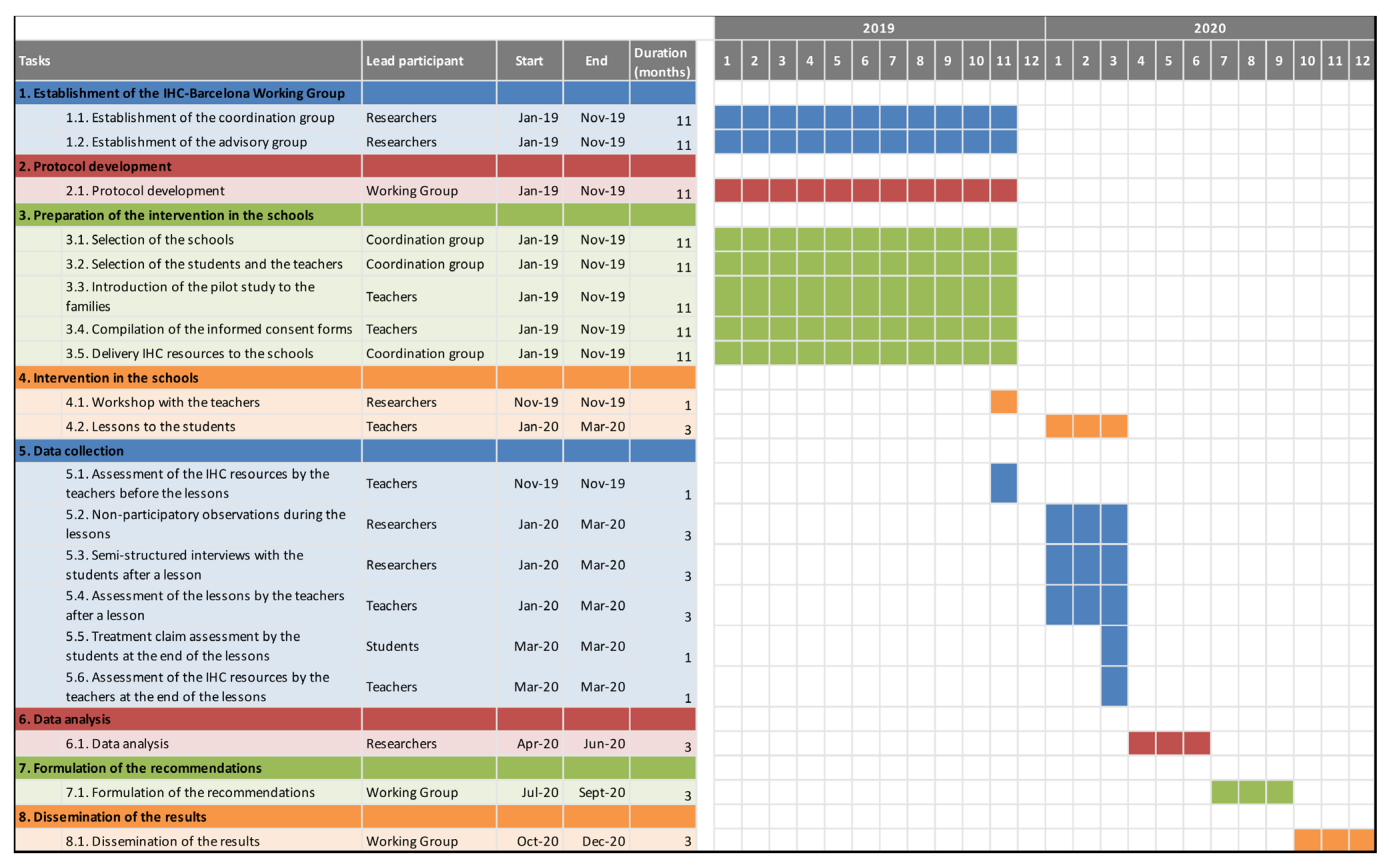
Gantt chart of the pilot study.

Figure 2 is a Gantt chart illustrating the schedule of the pilot study. Currently, we have started the intervention in schools with the teachers’ workshop.

## Discussion

It is important that people learn how to think critically about their health and how to make informed choices. The IHC project tackles this challenge from an innovative perspective because: 1) it focuses in children and 2) uses learning resources designed and assessed to facilitate the teaching and learning process. By introducing the IHC resources in a new context, we hope to contribute to the global effort to help people make informed choices regarding their health.

### Our study in the context of current knowledge

Introducing the IHC resources in schools can be considered as a health promotion and education intervention ^36^. According to the World Health Organization (WHO), the concept of health promotion comprises “the process of enabling people to increase control over, and to improve their health” ^37^. Additionally, health education comprises “consciously constructed opportunities for learning involving some form of communication designed to improve health literacy, including improving knowledge, and developing life skills which are conducive to individual and community health” ^37^. Health education and promotion interventions in schools have proven to be beneficial for the health of the population ^38, 39^.

There are several definitions of critical thinking, as well as several strategies to teach how to think critically ^9, 40^. In 1990, a Delphi panel of experts defined this ability as a “purposeful, self-regulatory judgment which results in interpretation, analysis, evaluation, and inference, as well as explanation of the evidential, conceptual, methodological, criteriological, or contextual considerations upon which that judgment is based” ^41^. Therefore, promoting critical thinking at schools can be not only useful in the health area ^22^, but also in other curricular areas (e.g., Mathematics, Science, Literacy) ^42^.

The IHC project offers several learning resources that were created accurately and explicitly, and have been assessed in a cluster randomised trial ^14, 22^. Thus far, there are few studies that assess the effect of the learning resources when acquiring competences ^43^. Moreover, the available studies show that the evaluated textbooks provide little learning support ^44– 46^. We must start demanding the same standards for evaluating educational interventions that are used for evaluating health interventions ^47^.

### Study strengths and limitations

Our proposal has several strengths. Firstly, before this study, we have translated the IHC resources into Spanish. A translator, researchers, students, teachers, and medical doctors participated in the translation process and fit the text of the IHC resources to this context. Secondly, we have expanded the profile of the users of interest (researchers, teachers, paediatricians, student representatives, family representatives, education and health stakeholders, and translators) to establish the pilot study's multidisciplinary working group. Thirdly and lastly, we will pilot an intervention in the schools that has already been shown to be effective in a cluster randomised trial in Uganda, where over 100 schools participated (100 teachers and 10,000 children).

Our proposal also has some limitations. The main limitation is using a convenience sample (small, geographically limited, and non-representative sample). However, we will provide a detailed description of our data collection and research context to help other stakeholders consider transferring our results to their settings. It is also worth noting that we will not be assessing the impact of the IHC resources in this study; due to this, we will not include a control group and we will not have a questionnaire validated to assess treatment claims for students.

### Implications for practice and research

The next contextualization activities will be: 1) content adaptation - if needed, 2) context analysis (exploring factors that can impact scaling up), and 3) validation of the CLAIM questionnaire test into Spanish for use in this context. The findings of the contextualization activities could inform the design of a cluster-randomised trial, to determine the effectiveness of the IHC resources in this context prior to scaling-up its use.

## Data availability

### Underlying data

No data are associated with this article.

### Extended data

Extended data is available at: https://figshare.com/articles/IHC_BCNPilotStudy/12221189/1 DOI: https://doi.org/10.6084/m9.figshare.12221189.v1 ^31^. Data are available under the terms of the Creative Commons Attribution 4.0 International license (CC-BY 4.0).
