# Supplementary material for: Learning to make informed health choices: Protocol for a pilot study in schools in Barcelona
Source: F1000Res. 2020 Jun 15;8:2018. Originally published 2019 Nov 28. [Version 3] doi: 10.12688/f1000research.21292.3 (PMC7268153; doi:10.12688/f1000research.21292.3)
Supplement: Peer review history

# Peer review: Reviewer response for version 2

**DOI:** 10.5256/f1000research.26302.r63344

**Published:** 2020-06-02

**Authors:** Loai Albarqouni

**Affiliations:** 1 The Institute for Evidence-Based Healthcare, Faculty of Health Sciences and Medicine, Bond University, Gold Coast, Qld, Australia

Thanks for this opportunity to review this revised manuscript. The authors have revised the manuscript taking into considerations the raised comments. No further comments on this protocol.

Is the study design appropriate for the research question?

Yes

Is the rationale for, and objectives of, the study clearly described?

Yes

Are sufficient details of the methods provided to allow replication by others?

Partly

Are the datasets clearly presented in a useable and accessible format?

Not applicable

Reviewer Expertise:

Evidence-based practice

I confirm that I have read this submission and believe that I have an appropriate level of expertise to confirm that it is of an acceptable scientific standard.

# Peer review: Reviewer response for version 2

**DOI:** 10.5256/f1000research.26302.r63343

**Published:** 2020-06-02

**Authors:** Luis Gabriel Cuervo

**Affiliations:** 1 Pan American Health Organization, Washington, DC, USA

Thanks for providing a revised version with substantial improvements including enhanced details and a Gantt Chart, the revised version ^1^ is an improvement over the original ^2^.

 The authors need to clarify if this protocol if for an ongoing study or the protocol they intend to implement. Once this has been clarified, the text needs to be amended accordingly. Currently, parts are written in the future tense (abstract, methods, most of the article), parts in the past tense (e.g. acknowledgments), and the implementation dates suggest that they study is way underway (2019-2020 School year, Gantt Chart). That some of the recommendations in the earlier review were not addressed also suggests that perhaps they were received after the fact.

 Given the disruption COVID-19 has brought, including to the school systems in Spain, it makes sense to point out if the implementation is/was affected and how this will affect the protocol.

 The paragraph entitled “Formulation of the recommendations” needs to be copy edited for clarity and grammar.

 Congratulations on your protocol for this important study, I wish you success with the prompt publication of the findings, and the implementation and scaling up of the intervention in Barcelona and beyond, as appropriate.

 Developing effective critical thinking skills from young ages seems increasingly important and I commend the authors for building on existing knowledge to further advance and expand its implementation. Well done!

Is the study design appropriate for the research question?

Partly

Is the rationale for, and objectives of, the study clearly described?

Partly

Are sufficient details of the methods provided to allow replication by others?

Partly

Are the datasets clearly presented in a useable and accessible format?

Not applicable

Reviewer Expertise:

Clinical Epidemiology and Biostatistics, Family Medicine, Research for Health, Policies on Research for Health, Health Systems Research, Public Health

I confirm that I have read this submission and believe that I have an appropriate level of expertise to confirm that it is of an acceptable scientific standard.

## References

[1] : Learning to make informed health choices: Protocol for a pilot study in schools in Barcelona. *F1000Research*.2020;8: 10.12688/f1000research.21292.2 10.12688/f1000research.21292.2 PMC726815332528654

[2] : Learning to make informed health choices: Protocol for a pilot study in schools in Barcelona. *F1000Research*.2019;8: 10.12688/f1000research.21292.1 10.12688/f1000research.21292.1 PMC726815332528654

# Response

**Published:** 2020-06-09

**Authors:** Laura Martínez García

**Affiliations:** Iberoamerican Cochrane Centre - Sant Pau Biomedical Research Institute (IIB-Sant Pau), Spain

**Comment 1 - Ongoing study**

**Response 1**

 We already included the state of the study in the “Study status” section; the text reads: “Figure 2 is a Gantt chart illustrating the schedule of the pilot study. Currently, we have started the intervention in schools with the teachers’ workshop”. We have reviewed the verb tense consistency. According to study status, we wrote the text in the future tense, except the “Introduction”, “Objectives”, and “Acknowledgements” sections.

**Comment 2 - COVID-19**

**Response 2**

 The first version of the “Learning to make informed health choices: Protocol for a pilot study in schools in Barcelona” was published on 28 Nov 2019. We will describe details about adjustments to the COVID19 in the publication of the results of the pilot study.

**Comment 3 - Formulation of the recommendations**

**Response 3**

 We have modified the text in the “Methods - Formulation of the recommendations” section according to the suggestion of the reviewers. The text reads: “The IHC-Barcelona Working Group will discuss the most relevant results from the qualitative analysis. They will consensus the potential changes of the IHC resources (dramatic changes, major changes, or minor changes) (Table 4). Finally, they will suggest and agree on recommendations - both for practice and research purposes - on how to use, how to adapt (if needed), and how to implement the IHC resources to this context.”

# Peer review: Reviewer response for version 1

**DOI:** 10.5256/f1000research.23446.r61277

**Published:** 2020-03-26

**Authors:** Luis Gabriel Cuervo, Sofía Giraldo-Hoyos

**Affiliations:** 1 Pan American Health Organization, Washington, DC, USA; 2 Pan American Health Organisation, Washington, DC, USA

We congratulate the authors for developing this protocol for a pilot study to adapt a tested intervention with a lasting effect in education of benefit to children, teachers and families. This is especially important at a time when pseudoscience and false statements frequently confuse people. There is great potential and value in people being able to critically assess information and distinguish valid information from unreliable one. Well done.

 We were pleased to review this protocol and to provide recommendations to enhance it ahead of the study.

**Is the rationale for, and objectives of, the study clearly described?**

 The manuscript provides a detailed description of the study previously done in Uganda ^1^, ^2^, ^3^ and needs to provide a similar level of detail of the protocol to be implemented in Barcelona.  The rationale and objectives are described under "Contextualization of the Informed Health Choices project" yet the descriptions need to provide in-depth detail of how the pilot is to be prepared, implemented, analysed, reported and used to inform future developments. Although it is a pleasure to read the description they do of the above cited research conducted in Uganda, if length were an issue, the authors can summarize some details by citing the studies and instead offering more details from their own study.

 The objectives need to explicitly state their intent to *to assess the effects of the intervention and replicability of the study in a IHC setting in a Spanish speaking country*, or *to prepare the IHC project for its scaling up in Barcelona, Spain*. This appears to be a central objective to the study.  Also describe their aims regarding the scaling-up of the intervention beyond the pilot schools.

 The information in the abstract needs to be matched to the key elements of the study, thus highlighting the ultimate purpose of the pilot and subsequent studies.

**Is the study design appropriate for the research question?**

 Please describe the target and study population in detail elaborating on the characteristics of the school districts and system in Barcelona and provide descriptive details of the target population (children, teachers, parents) disaggregating the information where possible by gender and other relevant variables (e.g. socioeconomic stratum, ethnicity, religion, etc.) ^4^, ^5^, ^6^. This way it will be clear if the pilot relates to the target population and/or elicit any limitations that need to be considered in the analysis or conclusions, as well as considerations for subsequent research.

 Elaborate on the criteria they will be using to select the participating schools in the convenience sample.

 The sample of teachers will be small thus it is important to illustrate any efforts to determine if these are representative of the broader population of teachers, to deal with imprecision or bias arising from the small sample, and to elaborate on considerations for their subsequent implementation research. How will they control if the selected teachers are over or under performers?

 We believe there are important conceptual issues to address under Ethical considerations. First, the decision to waive an ethical review falls with the Ethics Committee; apparently this was the case. However, we have some concerns with the rationale presented:

1. Describe what review process is being undertaken to ensure that their is compliance with local laws, with the assent of children (if appropriate), and whether the informed consent process (and corresponding documents) are appropriate for each of the groups of participants so that there is an informed decision;
2. Elaborate on the processes to protect the privacy of participants considering that this is a small sample size making feasible that identities can be exposed;
3. The rationale for the exemption is flawed because this is research in human subjects. If there was a reason for conducting an expedited review (ideal) or waiving it would be this being a “less than minimal risk” or “minimal risk study with the prospect of direct benefit to individual subjects”. Definitions are provided and surely there is equivalent European regulation that applies. ^4^, ^5^.

Clarify what the management will be if a student, teacher or parent decline to participate, considering the whole grade is participating. Will informed consent be managed at the individual or class level?    

**Are sufficient details of the methods provided to allow replication by others?**

 There are aspects that need more detail or perhaps a visual aid, such as:

- Specify the times when measurements will be done illustrating them with a PERT Chart complementing their timetable (or GANTT). This will make the information clear and also help to determine if the evaluations will be done at an appropriate time, avoiding for example these extending into the holiday season.
- Describe how the sample was chosen for the pilot and what implications this may have in the preparation of the larger implementation study.
- Details of the specific skills sought in the IHC-Barcelona Working Group and the Advisory Group are missing. It is of relevance to know what skills and attitudes are sought in the advisory group members to perform a proper job, and how they will be assessed. What criteria will be used in selecting the researchers and experts from the institutions listed? Will education policy makers be involved from the outset (advisable) and what positions do they have in Barcelona’s education system? Kindly describe what is the Escola Nova 21 initiative and how it relates to Barcelona’s education structure and standards. Elaborate on the implications -including bias that can result from the selection, and how this will be controlled.
- Describe what efforts will be made to determine the replicability and adaptations needed for the study in different settings in Barcelona and beyond. 
- Clarify the criteria that will be used to select the convenience sample as well the the justification for the sample size. Describe the process to be followed in selecting the schools meeting criteria 1, 2 and 3, and how these schools represent the broader education district of Barcelona, and the population of students. Elaborate how you will analyse representation.
- Provide specific dates for data collection and their place within the school calendar.
- Clarify if the selected classes will be co-ed (see note on PROGRESS) and if groups turn out to have >25 students, how will this be managed.

**Are the data sets clearly presented in a usable and accessible format?**

- The data analysis section would benefit from a table with the variables that will be analysed, and the analysis strategies (e.g. summary statistics, dispersion assessment, etc.) for each variable.
- Consider ways to minimize the variability of the assessments from teachers.
- Consider having double data capturing and whether and the thought process behind having one  researcher identifying, codifying and summarizing feedback instead of having a duplicate data-capturing with a third person as tie breaker, or other methods to enhance precision and assess consistency (page 10.)
- In section “Dissemination of the results” elaborate on the engagement and appropriation by education and science authorities as a strategy to enhance implementation and sustainability in Barcelona and beyond. Elaborate on the participation (if any) of science communicators and journalists, and potential advocates for education.
- Elaborate on the inclusion and exclusion criteria under “User participation” and describe the characteristics and more detailed relevant information of the group.
- In section “Discussion” highlight any preparations to adjust to the COVID19 (and if this may provide suitable material) and if possible frame the study on relevant policies that promote critical appraisal learning and partnerships with civil society to increase the value and use of research for health. We list a couple of policy documents and surely the authors can illustrate some local and European policies that apply as well ^6^, ^7^

We were delighted to read this study and how it will build on the success of a previous study to facilitate the implementation of an effective intervention in a different setting. Congratulations to the authors! It is important for these new studies to uphold the methodological rigour of the original studies and we have made some recommendations towards this end. A significant part of the protocol elaborates on the earlier studies referenced and some additional enhancements and details are needed to level this protocol with previous studies. We have provided actionable recommendations and we look forward to a revised version. This study has a tremendous potential to address with a key population, the current crisis that pseudo-science, false news, and misleading claims have brought upon societies, and we believe it needs to be advanced reported and developed in collaboration with education and science and technology leaders, seeking to strengthen appropriation and uptake. We also believe that equity considerations need to be reflected in every aspect of this study, and we have made some recommendations towards this. Well done, we look forward to a revised version!

Is the study design appropriate for the research question?

Partly

Is the rationale for, and objectives of, the study clearly described?

Partly

Are sufficient details of the methods provided to allow replication by others?

Partly

Are the datasets clearly presented in a useable and accessible format?

Not applicable

Reviewer Expertise:

NA

We confirm that we have read this submission and believe that we have an appropriate level of expertise to confirm that it is of an acceptable scientific standard, however we have significant reservations, as outlined above.

## References

[1] : Informed health choices intervention to teach primary school children in low-income countries to assess claims about treatment effects: process evaluation. *BMJ Open*.2019;9(9) : 10.1136/bmjopen-2019-030787 10.1136/bmjopen-2019-030787 PMC674765431511291

[2] : Effects of the Informed Health Choices primary school intervention on the ability of children in Uganda to assess the reliability of claims about treatment effects, 1-year follow-up: a cluster-randomised trial. *Trials*.2020;21(1) : 10.1186/s13063-019-3960-9 27 10.1186/s13063-019-3960-9 31907013PMC6945419

[3] : Effects of the Informed Health Choices primary school intervention on the ability of children in Uganda to assess the reliability of claims about treatment effects: a cluster-randomised controlled trial. *The Lancet*.2017;390(10092) : 10.1016/S0140-6736(17)31226-6 374-388 10.1016/S0140-6736(17)31226-6 28539194

[4] : Standards and operational guidance for ethics review of health-related research with human participants. Reference source 26269877

[5] Electronic Code of Federal Regulations, Part 46—Protection of Human Subjects. Reference source 11660819

[6] : The WHO strategy onresearch for health. Reference source

[7] Pan American Health Organisation World Health Organisation 49th Directing Council and 61st Session of the Regional Committee of WHO. Reference source

# Response

**Published:** 2020-05-01

**Authors:** Laura Martínez García

**Affiliations:** Iberoamerican Cochrane Centre - Sant Pau Biomedical Research Institute (IIB-Sant Pau), Spain

**Comment 1.1 - Detailed description of the study**

**Response 1.1**

 We assume that the details requested by the reviewers in this comment are disaggregated below. We describe our responses to each of the comments below.

**Comment 1.2 - Objectives**

**Response 1.2**

 The primary objective of the pilot study is to “explore the students’ and teachers’ experience when using the learning resources of the IHC project in the context of Barcelona (Spain).” To achieve this objective, we will conduct a pilot study in a convenience sample of three schools in Barcelona. Our principal results will be recommendations - both for practice and research - on how to use, how to adapt (if needed), and how to implement the IHC resources in this context.

 To evaluate the effect of the intervention, we would have to conduct a cluster-randomised trial [Nsangi 2017]. We already described this limitation in the “Discussion - Study strengths and limitations” section; the text reads “It is also worth noting that we will not be assessing the impact of the IHC resources in this study; due to this, we will not include a control group and we will not have a questionnaire validated to assess treatment claims for students.”

**Comment 1.3 - Scaling-up the intervention**

**Response 1.3**

 We have modified the text in the “Discussion - Implications for practice and research” section according to the suggestion of the reviewers. The text reads: “The next contextualization activities will be: 1) content adaptation - if needed, 2) context analysis (exploring factors that can impact scaling up), and 3) validation of the CLAIM questionnaire test into Spanish for use in this context. The findings of the contextualization activities could inform the design of a cluster-randomised trial, to determine the effectiveness of the IHC resources in this context prior to scaling-up its use.”.

**Comment 1.4 - Abstract**

**Response 1.4**

 We have modified the text in the “Abstract” section according to the suggestion of the reviewers. The text reads: “The findings of the contextualization activities could inform the design of a cluster-randomised trial, to determine the effectiveness of the IHC resources in this context prior to scaling-up its use.”.

**Comment 2.1 - Study population**

**Response 2.1**

 We have included, as extended data, the description of the intervention in the schools using TIDieR checklist. The text now reads “The intervention in the schools will include: 1) a workshop with the teachers, and 2) lessons to the students (Extended data 4 provides a description of the intervention using the TIDieR checklist) [Hoffmann 2014, Martínez García 2020].”.

 We will describe the study population (children, teachers, parents) and relevant related variables (e.g. gender, socioeconomic stratum, ethnicity, or religion, etc.) in the publication of the results of the pilot study.

**Comment 2.2 - Eligibility criteria of the sample**

**Response 2.2**

 Relating the eligibility criteria of the sample, please see “Response 2” for Reviewer 1.

**Comment 2.3 - Generalisation and implementation considerations**

**Response 2.3**

 The primary objective of the pilot study is to “explore the students’ and teachers’ experience when using the learning resources of the IHC project in in the context of Barcelona (Spain).” To achieve this objective, we will conduct a pilot study in a convenience sample of three schools in Barcelona. Our principal results will be recommendations - both for practice and research - on how to use, how to adapt (if needed), and how to implement the IHC resources in this context.

 To consider to generalise the results, we would have to include a representative sample [Nsangi 2017]. We have included this limitation in the “Discussion - Study strengths and limitations” section; the text reads “The main limitation is using a convenience sample (small, geographically limited, and non-representative sample). However, we will provide a detailed description of our data collection and research context to help other stakeholders consider transferring our results to their settings.”.

 To evaluate implementation considerations, we would have to conduct a context analysis [IHC 2020]. The mail objectives of a context analysis would be: 1) explore what demand there is for learning resources for teaching critical thinking about health in schools, 2) map where teaching critical thinking about health best fits in the curriculum, 3) identify and examine relevant resources already in use, and 4) explore conditions for introducing new learning resources [IHC 2020]. The development of context analysis in our setting is already considered in the “Discussion - Implications for practice and research” section, the text reads: “The next contextualization activities will be: 1) content adaptation - if needed, 2) context analysis, and 3) validation of the CLAIM questionnaire test into Spanish for use in this context. Finally, a cluster randomised trial with the adapted IHC resources could be conducted to evaluate their effect on the students’ ability to assess treatment claims in this context.”.

**Comment 2.4- Ethical considerations**

**Response 2.4**

 We have modified the text in the “Ethical considerations” section according to the suggestion of the reviewers. The text now reads: “The study protocol has obtained an approval exemption (does not include patients, biological specimens or clinical data) from the Ethics Committee of the Hospital de la Santa Creu i Sant Pau (Barcelona, Spain) [CEIm 2020]. We will inform participants about the pilot study and will request their written informed consent (Extended data 1-3) [Martínez García 2020]. If a family does not want to participate, the student will participate in the lessons as a curricular activity but will not participate in any of the study data collection activities. We will anonymise the data removing participant and school name.”

**Comment 3.1 - Gantt chart**

**Response 3.1**

 We have modified the text and Figure 2 according to the suggestion of the reviewers. The text now reads “Figure 2 is a Gantt chart illustrating the schedule of the pilot study.”

**Comment 3.2 - Eligibility criteria of the sample**

**Response 3.2**

 Relating the eligibility criteria of the sample, please see “Response 2” for Reviewer 1.

 Relating the implementation considerations, please see “Response 2.3” above.

**Comment 3.3 - IHC-Barcelona Working Group**

**Response 3.3**

 To establish the IHC-Barcelona Working Group, we only considered profession criteria (researchers, teachers, paediatricians, education and health stakeholders, and translators) or family-related criteria (student and family representatives). We will describe detail about IHC-Barcelona Working Group in the publication of the results of the pilot study.

**Comment 3.4 - Escola Nova 2**

**Response 3.4**

 Relating the eligibility criteria of the sample, please see “Response 2” for Reviewer 1.

**Comment 3.5 - Contextualising IHC resources**

**Response 3.5**

 The primary objective of the pilot study is to “explore the students’ and teachers’ experience when using the learning resources of the IHC project in the context of Barcelona (Spain).” To achieve this objective, we will conduct a pilot study in a convenience sample of three schools in Barcelona. Our principal results will be recommendations - both for practice and research - on how to use, how to adapt (if needed), and how to implement the IHC resources in this context.

 The IHC project team has developed several guidelines for contextualising IHC resources [IHC 2019a, IHC 2019b, IHC 2017a, IHC 2017b]. Different working groups from more than 20 countries are adapting, or planning adaptation, of the IHC resources to their context [IHC Newsletter 2019, IHC Newsletter 2020].

**Comment 3.6 - Sample and eligibility criteria**

**Response 3.6**

 Relating the sample and eligibility criteria, please see “Response 2” for Reviewer 1.

**Comment 3.7 - Gantt chart**

**Response 3.7**

 The schedule of the pilot study is illustrated in Figure 2. We will describe detail about data collection in the publication of the results of the pilot study.

**Comment 3.8 - Study population**

**Response 3.8**

 We will describe detail about co-educational classes or the number of students in the publication of the results of the pilot study.

**Comment 4.1 - Study variables**

**Response 4.1**

 The variables of the pilot study are already listed in “Table 3. Pilot study variables”. The reviewers' proposal “to include a data analysis plan” [Simpson 2015] is not a request of the F1000Research journal [https://f1000research.com/for-authors/article-guidelines/study-protocols].

**Comment 4.2 - Variability of the assessments**

**Response 4.2**

 We will use ad hoc questionnaires and guides to collect the data to minimize the variability of the assessments. We included questionnaires and guides as extended data, available at: https://figshare.com/articles/IHC_BCNPilotStudy/12221189/1.

**Comment 4.3 - Data collection**

**Response 4.3**

 We have included a review process to improve the precision of the data that we will collect from “Non-participatory observations during the lessons” and “Semi-structured interviews with the students after a lesson”. The included text reads: “Another researcher will check the notes with the recorded audios. The two researchers will resolve potential disagreements by discussion, and if necessary, by consulting a third researcher.”

**Comment 4.4 - Dissemination of the results**

**Response 4.4**

 We already included education and science authorities in the “Dissemination of the results” section; the text reads: “The dissemination activities of the pilot study results will include: 1) publication in a peer-reviewed journal, 2) publication in several Internet resources (for example, related web pages, electronic bulletins and social media), and 3) introduction to the different users of interest (researchers, teachers, paediatricians, student representatives, family representatives, education and health stakeholders, and translators) in conferences, workshops and meetings.”.

 Relating the implementation considerations, please see “Response 2.3” above.

**Comment 4.5 - IHC-Barcelona Working Group**

**Response 4.5**

 Relating the description of IHC-Barcelona Working Group, please see “Response 3.3” above.

**Comment 4.6 - COVID19**

**Response 4.6**

 The first version of the “Learning to make informed health choices: Protocol for a pilot study in schools in Barcelona” was published on 28 Nov 2019 [Martínez García 2019]. We will describe detail about preparations to adjust to the COVID19 in the publication of the results of the pilot study.

**References**

- [CEIm 2020] Ethics Committee for research with medicinal products (CEIm). 2020 [online]. Available at: http://www.recercasantpau.cat/en/clinical-research/ceic/. [Accessed April 16, 2020].
- [Hoffmann 2014] Hoffmann TC, Glasziou PP, Boutron I, Milne R, Perera R, Moher D, et al. Better reporting of interventions: template for intervention description and replication (TIDieR) checklist and guide. BMJ. 2014;348:g1687.
- [IHC 2017a] The Informed Health Choices group. Guide for piloting the Informed Health Choices (IHC) learning resources. IHC Working Paper, Norwegian Institute of Public Health, 2017.
- [IHC 2017b] The Informed Health Choices group. Guide for translating and adapting the Informed Health Choices (IHC) podcast. Informed Health Choices Working Paper, Norwegian Institute of Public Health, 2017.
- [IHC 2019a] The Informed Health Choices group. Guide for translating the Informed Health Choices school resources. Informed Health Choices Working Paper, Norwegian Institute of Public Health, 2019.
- [IHC 2019b] The Informed Health Choices group. Resource production guide for translating and adapting Informed Health Choices learning resources. Informed Health Choices Working Paper, Norwegian Institute of Public Health, 2019.
- [IHC 2020] IHC CHOICE team. Protocol for a context analysis: Exploring the considerations for introducing digital learning resources for critical thinking about health in secondary schools. Informed Health Choices Working Paper, Norwegian Institute of Public Health, 2020.
- [IHC Newsletter 2019] The Informed Health Choices Group. Informed Health Choices Newsletter 2019 Norwegian Institute of Public Health. 2019 [online]. Available at: https://www.informedhealthchoices.org/wp-content/uploads/2019/03/IHC-Newsletter-2019.pdf [Accessed April 16, 2020].
- [IHC Newsletter 2020] The Informed Health Choices Group. Informed Health Choices Newsletter 2020. Norwegian Institute of Public Health. 2020 [online]. Available at: https://www.informedhealthchoices.org/wp-content/uploads/2020/02/2020-Informed-Health-Choices-newsletter-1.pdf. [Accessed April 16, 2020].
- [Martínez García 2019] Martínez García L, Alonso-Coello P, Asso Ministral L, Ballesté-Delpierre C, Canelo Aybar C, de Britos C, et al. Learning to make informed health choices: Protocol for a pilot study in schools in Barcelona [version 1; peer review: awaiting peer review]. F1000Research. 2020 (https://f1000research.com/articles/8-2018).
- [Martínez García 2020] Martínez García L. IHC@BCNPilotStudy [Internet]. figshare; 2020. Available at: https://figshare.com/articles/IHC_BCNPilotStudy/12221189/1 [Accessed April 30, 2020].
- [Nsangi 2017] Nsangi A, Semakula D, Oxman AD, Austvoll-Dahlgren A, Oxman M, Rosenbaum S, *et al*. Effects of the Informed Health Choices primary school intervention on the ability of children in Uganda to assess the reliability of claims about treatment effects: a cluster-randomised controlled trial. Lancet. 2017;390(10092):374-388.
- [Simpson 2015] Simpson SH. Creating a Data Analysis Plan: What to Consider When Choosing Statistics for a Study. Can J Hosp Pharm. 2015; 68(4): 311–317.

# Peer review: Reviewer response for version 1

**DOI:** 10.5256/f1000research.23446.r58609

**Published:** 2020-02-10

**Authors:** Loai Albarqouni

**Affiliations:** 1 The Institute for Evidence-Based Healthcare, Faculty of Health Sciences and Medicine, Bond University, Gold Coast, Qld, Australia

Thanks for inviting me to review this protocol. The author proposes a pilot study to evaluate the IHC learning resources in 3 primary schools in Barcelona. the protocol is well -designed and -written. Few comments below:

**Introduction **

- I would cite a few references to support each of the 5 arguments for starting the IHC project in primary school.

**Methods**

- I would justify the sampling methods & eligibility criteria of the schools and describe if these 3 schools are representatives to the average school in Barcelona (e.g. a number of students).
- I would consider TIDier Checklist to better describe intervention details.
- I would describe the time points for each of the outcome measures.
- Data analysis – ‘qualitative’ variables should be replaced by ‘categorical’ and ‘quantitative’ variables by ‘continuous’ – Both quantitative measures.
- Authors should describe whether the resources have been translated and validated into Spanish.

Is the study design appropriate for the research question?

Yes

Is the rationale for, and objectives of, the study clearly described?

Yes

Are sufficient details of the methods provided to allow replication by others?

Partly

Are the datasets clearly presented in a useable and accessible format?

Not applicable

Reviewer Expertise:

Evidence-based practice

I confirm that I have read this submission and believe that I have an appropriate level of expertise to confirm that it is of an acceptable scientific standard, however I have significant reservations, as outlined above.

# Response

**Published:** 2020-05-01

**Authors:** Laura Martínez García

**Affiliations:** Iberoamerican Cochrane Centre - Sant Pau Biomedical Research Institute (IIB-Sant Pau), Spain

**Comment 1 - References in the introduction**

****

**Response 1**

 We have included relevant references to support the reasons for starting the IHC project in primary school children. The text now reads: “There are several reasons the IHC project started with primary school children: 1) children can learn about fair comparisons (controlled research) and critical appraisal (in some countries, teaching these basic capabilities is already part of the curriculum) [Oxman 2020, START 2020]; 2) primary school interventions can reach a large population group, before many of them leave school [UNESCO 2019]; 3) compared to adults, children have more time to learn and show less resistance to change with regard to their beliefs, attitudes or behaviours [Vosniadou 2013]; 4) teaching children to think critically improves their academic performance [Oxman 2019]; and 5) learning how to think critically about claims about treatment effects can help them, once they become adults, to make decisions about their health and to contribute, as citizens or as health decision-makers, to develop and implement health policies [Nsangi 2017].”

**Comment 2 - Sample, eligibility criteria, and description of the schools**

****

**Response 2**

**Sample **We have included a relevant reference to justify the sampling. The text now reads: “To achieve the objective, we will select a convenience sample of three schools in Barcelona [Etikan 2016].” On the other hand, we already highlight this limitation in the “Discussion - Study strengths and limitations” section, the text reads: “The main limitation is using a convenience sample (small, geographically limited, and non-representative sample).”

 ​​​ **Eligibility criteria **We have clarified that the IHC-Barcelona Working Group made a consensus to establish the eligibility criteria of the sample. The text now reads: “The IHC-Barcelona Working Group reach a consensus on eligibility criteria of the schools: 1) schools included in the school directory from the Department of Education from the Government of Catalonia (2018-2019); 2) schools that have participated in a health promotion programme (2016-2017) [Salvador 2018]; and 3) schools that take part in the initiative Escola Nova 21 (alliance of schools and civil society institutions for an advanced education system, carried out between 2016 - 2019, and responding to United Nations and UNESCO’s call for the participation of all sectors in an inclusive process to make possible the education paradigm shift). We will also take into consideration whether the schools include students that are representative of the neighbourhood, if they are in different neighbourhoods of the city, and their type of funding (two public schools and one private or charter school).

**Description of the schools **We will describe the included schools and discuss their representativeness in the publication of the results of the pilot study.

**Comment 3 - TIDieR Checklist**

****

**Response 3**

 We have included, as extended data, the description of the intervention in the schools using TIDieR checklist. The text now reads “The intervention in the schools will include: 1) a workshop with the teachers, and 2) lessons to the students (Extended data 4 provides a description of the intervention using the TIDieR checklist) [Hoffmann 2014, Martínez García 2020]. Each of the activities is summarised below:”

 Also, we have combined all extended data in a single document to facilitate its availability to readers. The text in the "Data availability" section now reads: “Extended data is available at: https://figshare.com/articles/IHC_BCNPilotStudy/12167622 [Martínez García 2020].” We have modified the reference according “Martínez García L. IHC@BCNPilotStudy [Internet]. figshare; 2020. Available at: https://figshare.com/articles/IHC_BCNPilotStudy/12221189/1 [Accessed April 30, 2020].”

**Comment 4 - Time points of outcomes measures**

**Response 4**

 We have clarified the time points for data collection throughout the manuscript. The activities now read:

1. Assessment of the IHC resources by the teachers before the lessons
2. Non-participatory observations during the lessons
3. Semi-structured interviews with the students after a lesson
4. Assessment of the lessons by the teachers after a lesson
5. Treatment claim assessment by the students at the end of the lessons
6. Assessment of the IHC resources by the teachers at the end of the lessons

**Comment 5 - Data analysis**

****

**Response 5**

 We have amended the text, now it reads: “We will perform a descriptive analysis of the categorical variables (absolute and relative frequencies), and the continuous variables (mean and standard deviation or median and range).”

**Comment 6 - Translation and validation into Spanish of IHC resources**

**Response 6**

 We have included a relevant reference to support the method used to translate into Spanish the IHC resources. The text now reads “The working group from the Sant Pau Biomedical Research Institute (IIB Sant Pau) - Iberoamerican Cochrane Centre (CCIb) has translated the IHC resources into Spanish based on methods proposed by the IHC Working Group [IHC 2019].”

 On the other hand, we already highlight the strengths and limitations of translation process in the “Discussion - Study strengths and limitations” section, the text reads:

- Strength: “Firstly, before this study, we have translated the IHC resources into Spanish. A translator, researchers, students, teachers, and medical doctors participated in the translation process and fit the text of the IHC resources to this context.”
- Limitation: “It is also worth noting that we will not be assessing the impact of the IHC resources in this study; due to this, we will not include a control group and we will not have a questionnaire validated to assess treatment claims for students.”

**References**

- [Etikan 2016] Etikan I, Abubakar Musa S, Sunusi Alkassim R. Comparison of Convenience Sampling and Purposive Sampling. American Journal of Theoretical and Applied Statistics. 2016;5(1):1-4.
- [Hoffmann 2014] Hoffmann TC, Glasziou PP, Boutron I, Milne R, Perera R, Moher D, et al. Better reporting of interventions: template for intervention description and replication (TIDieR) checklist and guide. BMJ. 2014;348:g1687.
- [IHC 2019] The Informed Health Choices group. Guide for translating the Informed Health Choices school resources. Informed Health Choices Working Paper, Norwegian Institute of Public Health, 2019.
- [Martínez García 2020] Martínez García L. IHC@BCNPilotStudy [Internet]. figshare; 2020. Available at: https://figshare.com/articles/IHC_BCNPilotStudy/12221189/1 [Accessed April 30, 2020].
- [Nsangi 2017] Nsangi A, Semakula D, Oxman AD, Austvoll-Dahlgren A, Oxman M, Rosenbaum S, *et al*. Effects of the Informed Health Choices primary school intervention on the ability of children in Uganda to assess the reliability of claims about treatment effects: a cluster-randomised controlled trial. Lancet. 2017;390(10092):374-388.
- [Oxman 2020] Oxman A, Martínez García L. Comparison of the Informed Health Choices Key Concepts Framework to other frameworks relevant to teaching and learning how to think critically about health claims and choices: a systematic review [version 1; peer review: awaiting peer review]. F1000Research. 2020.
- [Salvador 2018] Salvador M, Bastida A, Martínez SN, Juárez O, Artazcoz L. Programes de promoció de la salut en centres d’educació primària i secundària de Barcelona. Avaluació del curs 2016-2017. Barcelona: Agència de Salut Pública de Barcelona; 2018.
- [START 2020] Health Research Board – Trials Methodology Research network (HRB-TMRN), National University of Ireland Galway. START – Schools Teaching Awareness of Randomised Trials [online]. Available at: https://www.hrb-tmrn.ie/public-engagement/start-competition [Accessed April 16, 2020]
- [UNESCO 2019] UNESCO Institute for Statistics. New Methodology Shows that 258 Million Children, Adolescents and Youth Are Out of School [online]. Available at: http://uis.unesco.org/sites/default/files/documents/new-methodology-shows-258-million-children-adolescents-and-youth-are-out-school.pdf [Accessed April 16, 2020]
- [Vosniadou 2013] Vosniadou S. International handbook of research on conceptual change. 2nd edition. Oxford: Routledge, 2013.
